# Supplementary material for: Conditional chemoconnectomics (cCCTomics) as a strategy for efficient and conditional targeting of chemical transmission
Source: eLife. 2024 Apr 30;12:RP91927. doi: 10.7554/eLife.91927 (PMC11060718; doi:10.7554/eLife.91927)
Supplement: Figure 3—source data 3. [file elife-91927-fig3-data3.zip › Figure 3 source data3 part 1.zip!/Figure 3-Source data 3-part1/1063-13-CRISPResso_Report_8cz_fsdr.zip!/CRISPResso_on_1063_16/3b.Insertion_deletion_substitutions_size_hist.pdf]

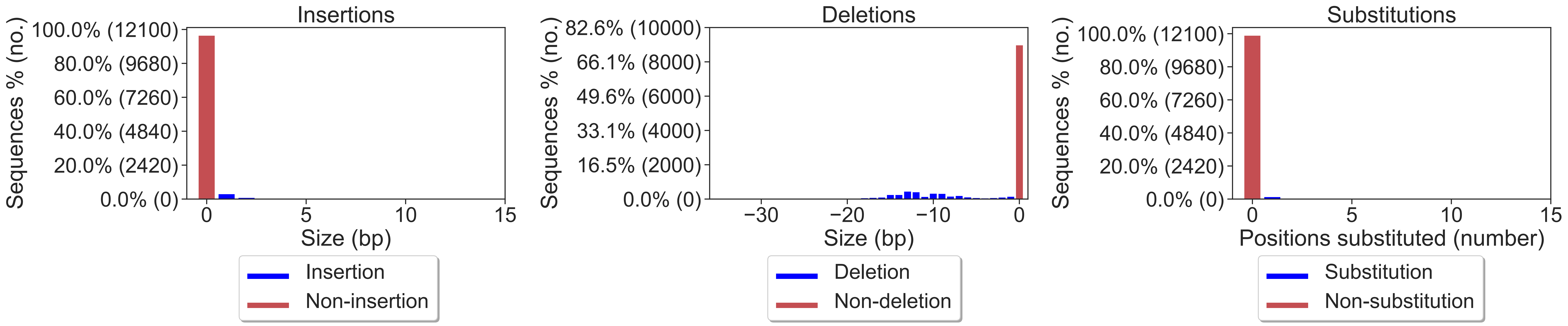
Insertions
Sequences % (no.)
100.0% (12100)
80.0% (9680)
60.0% (7260)
40.0% (4840)
20.0% (2420)
0.0% (0)
0
5
10
15
Size (bp)
Insertion
Non-insertion
Deletions
Sequences % (no.)
82.6% (10000)
66.1% (8000)
49.6% (6000)
33.1% (4000)
16.5% (2000)
0.0% (0)
−30
−20
−10
0
Size (bp)
Deletion
Non-deletion
Substitutions
Sequences % (no.)
100.0% (12100)
80.0% (9680)
60.0% (7260)
40.0% (4840)
20.0% (2420)
0.0% (0)
0
5
10
15
Positions substituted (number)
Substitution
Non-substitution
